# High-Flow Congenital Arteriovenous Malformation of the Posterior Chest Wall in a Young Adult: A Case Report on a Rare Anomaly

**DOI:** 10.7759/cureus.88279

**Published:** 2025-07-19

**Authors:** Ijan Dhamala, Soniya Bhatta, Dik P Katel, Manish Acharya, Krishnaprasad Bashyal

**Affiliations:** 1 Cardiothoracic and Vascular Surgery, Kathmandu Medical College, Kathmandu, NPL; 2 Anesthesia, All India Institute of Medical Sciences, Rishikesh, IND; 3 Radiodiagnosis, Institute of Medicine (IOM), Kathmandu, NPL

**Keywords:** arteriovenous malformation, chest wall mass, congenital vascular anomaly, embolization, surgical excision

## Abstract

Arteriovenous malformations (AVMs) are rare congenital vascular anomalies characterized by direct connections between arteries and veins without intervening capillary beds, resulting in high-flow dynamics. While AVMs commonly affect the craniofacial region and extremities, involvement of the chest wall is exceedingly rare. We describe the case of a 19-year-old male patient who had a painless, progressively enlarging swelling in the right posterior chest wall that had been present since birth. Magnetic resonance imaging revealed a large, high-signal lesion involving subcutaneous and muscular planes of the right posterior chest wall, while computed tomography angiography demonstrated arterial feeders arising from multiple intercostal arteries, confirming the diagnosis of a high-flow AVM. A multidisciplinary approach was employed, beginning with fluoroscopy-guided embolization using polyvinyl alcohol particles and Gelfoam to devascularize the lesion. The patient had a successful en bloc surgical excision performed under general anesthesia one week later. Postoperative recovery was uneventful, and no recurrence was noted at six-month follow-up. This case underscores the importance of a staged, image-guided, and multidisciplinary treatment strategy in managing large, high-flow AVMs in rare anatomical locations such as the chest wall.

## Introduction

Arteriovenous malformations (AVMs) are rare vascular anomalies that result from errors during embryogenesis, where arteries connect directly to veins, thereby bypassing capillary beds. These lesions are often congenital and can remain asymptomatic for years until exacerbated by trauma, hormonal changes, or increased physiological demand [1]. While AVMs are most frequently located in the head, neck, and extremities, those involving the chest wall are exceedingly rare, and the literature has only documented a small number of these cases [2,3].

High-flow AVMs, due to their significant vascularity, present a risk of complications such as spontaneous hemorrhage, high-output cardiac failure, and cosmetic deformity [4]. Imaging modalities, such as magnetic resonance imaging (MRI) and computed tomography (CT) angiography, are critical in delineating the lesion and planning treatment [5]. Management is often complex and necessitates a multidisciplinary approach, including preoperative embolization to reduce intraoperative blood loss, followed by surgical excision [6].

We present a rare case of a congenital high-flow AVM located in the right posterior chest wall of a young male, which was managed successfully with staged endovascular and surgical intervention.

## Case presentation

A 19-year-old male patient presented to our outpatient surgical unit with a longstanding, gradually enlarging swelling over the right posterior chest wall. According to the patient, the swelling had been present since birth but had increased significantly in size over the past two years. There was no associated pain, tenderness, or discoloration. He denied any episodes of bleeding, respiratory difficulty, or systemic symptoms. There was no history of vascular abnormalities in the family.

**Figure 1:**
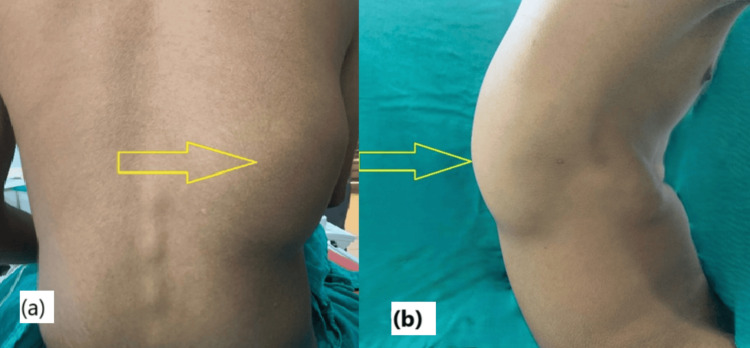
Preoperative clinical photographs showing a large, soft, nontender swelling over the right posterior chest wall. (a) Posterior view demonstrating the extent of the lesion across the lower thoracic region. (b) Lateral view showing the protrusion and soft-tissue fullness without skin discoloration or ulceration

On clinical examination, a large, soft, compressible mass was observed over the right posterolateral thorax. It extended inferiorly from approximately the level of the 9th to the 12th rib. Palpation revealed a palpable thrill, and auscultation over the mass demonstrated a continuous bruit, suggesting high vascular flow. The overlying skin was intact with no ulceration or discoloration (Figure 1).

No abnormalities were detected on systemic examination. Laboratory investigations were within normal levels (Table 1).

**Table 1:** Laboratory values of the case

Investigation	Observation result	Reference range
Hemoglobin	13.5 g/dL	13-17 g/dL
Platelet count	250,000/mm³	150,000-450,000/mm³
Total leukocyte count	7,800/mm³	4,000-11,000/mm³
Prothrombin time	12.5 seconds	11-13.5 seconds
International normalized ratio	1	0.9-1.2
Activated partial thromboplastin time	31 seconds	25-35 seconds
Serum creatinine	1 mg/dL	0.6-1.3 mg/dL
Erythrocyte sedimentation rate	12 mm/hour	0-20 mm/hour
C-reactive protein	2 mg/L	<5 mg/L

**Figure 2:**
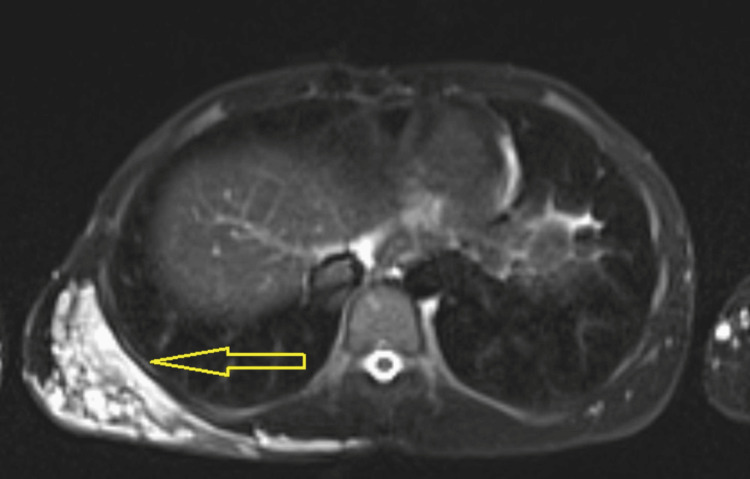
Axial T2-weighted MRI of the chest, demonstrating a large, hyperintense lesion in the right posterior chest wall with multiple dilated and tortuous vascular channels MRI: magnetic resonance imaging

MRI of the chest and abdomen revealed a high-signal intensity lesion in T1, T2, and short tau inversion recovery sequences, with multiple dilated, tortuous vascular channels measuring approximately 7.4 × 11.7 × 15.8 cm in anteroposterior, transverse, and craniocaudal dimensions, respectively. The lesion involved the subcutaneous, intramuscular, and intermuscular planes without extending into the thoracic cavity or bony erosion (Figure 2).

**Figure 3:**
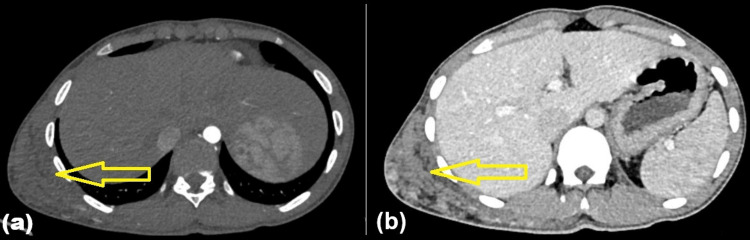
CECT scan of the chest, abdomen, and pelvis with angiography. (a) Axial CT angiography image, showing arterial supply to the chest wall lesion from multiple intercostal arteries. (b) Axial CECT scan, showing a large, ill-defined lobulated lesion involving the subcutaneous and muscular planes of the right posterior chest wall CT: computed tomography; CECT: contrast-enhanced computed tomography

An abdominal, pelvic, and chest contrast-enhanced computed tomography scan with angiography revealed a lobulated, ill-defined mass in the right chest wall. It enhanced heterogeneously and received arterial supply from the right 9th, 10th, 11th, and 12th intercostal arteries, consistent with a high-flow AVM (Figure 3).

**Figure 4:**
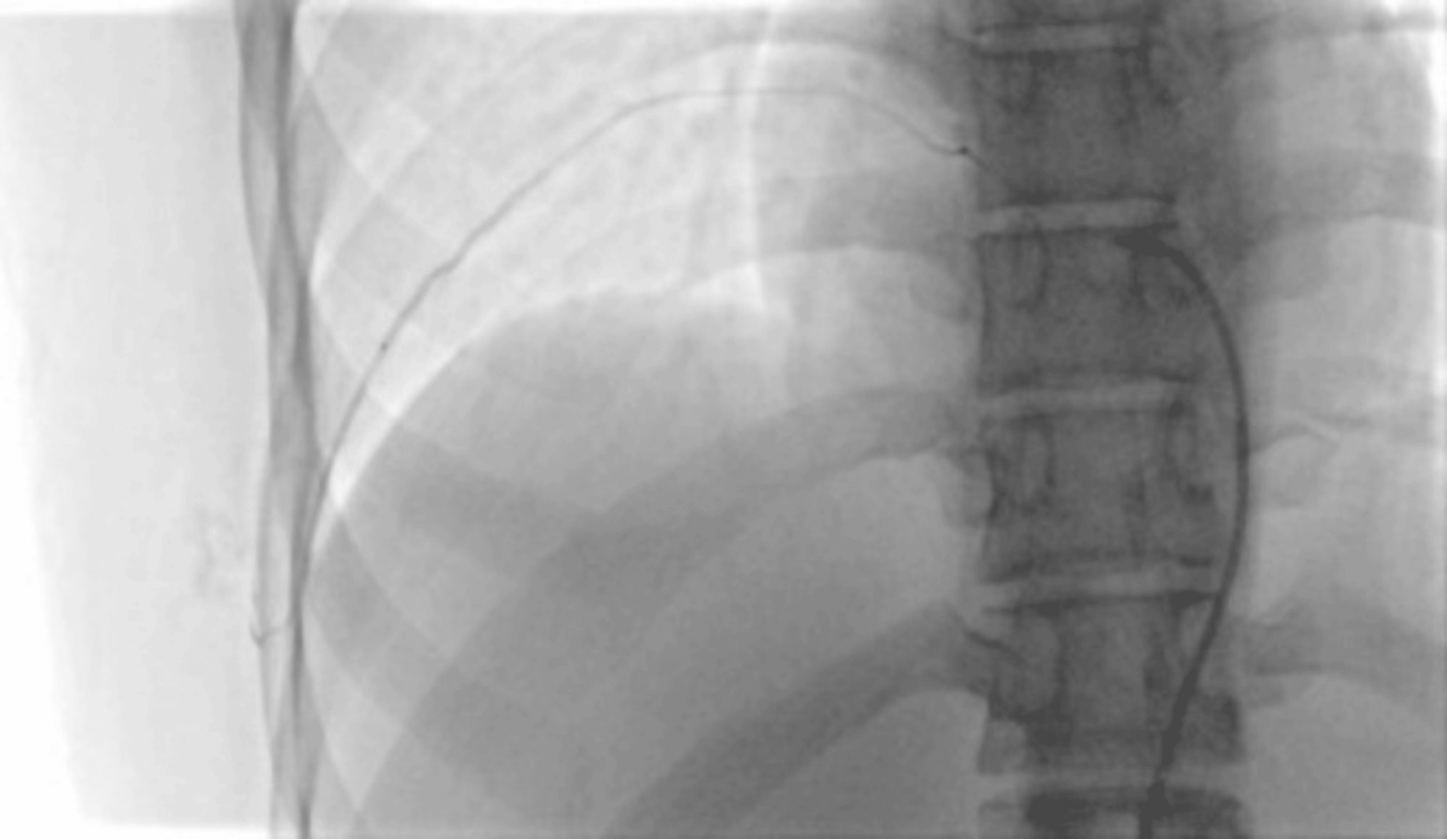
Fluoroscopic image during embolization showing catheterization of intercostal arteries supplying the arteriovenous malformation

Given the lesion’s size and vascularity, a multidisciplinary team comprising interventional radiology, thoracic surgery, and anesthesia recommended a two-stage approach. The patient underwent fluoroscopy-guided embolization using polyvinyl alcohol (PVA) particles (500-700 µm) and gelfoam slurry. Arterial feeders from the aforementioned intercostal arteries were successfully embolized (Figure 4).

**Figure 5:**
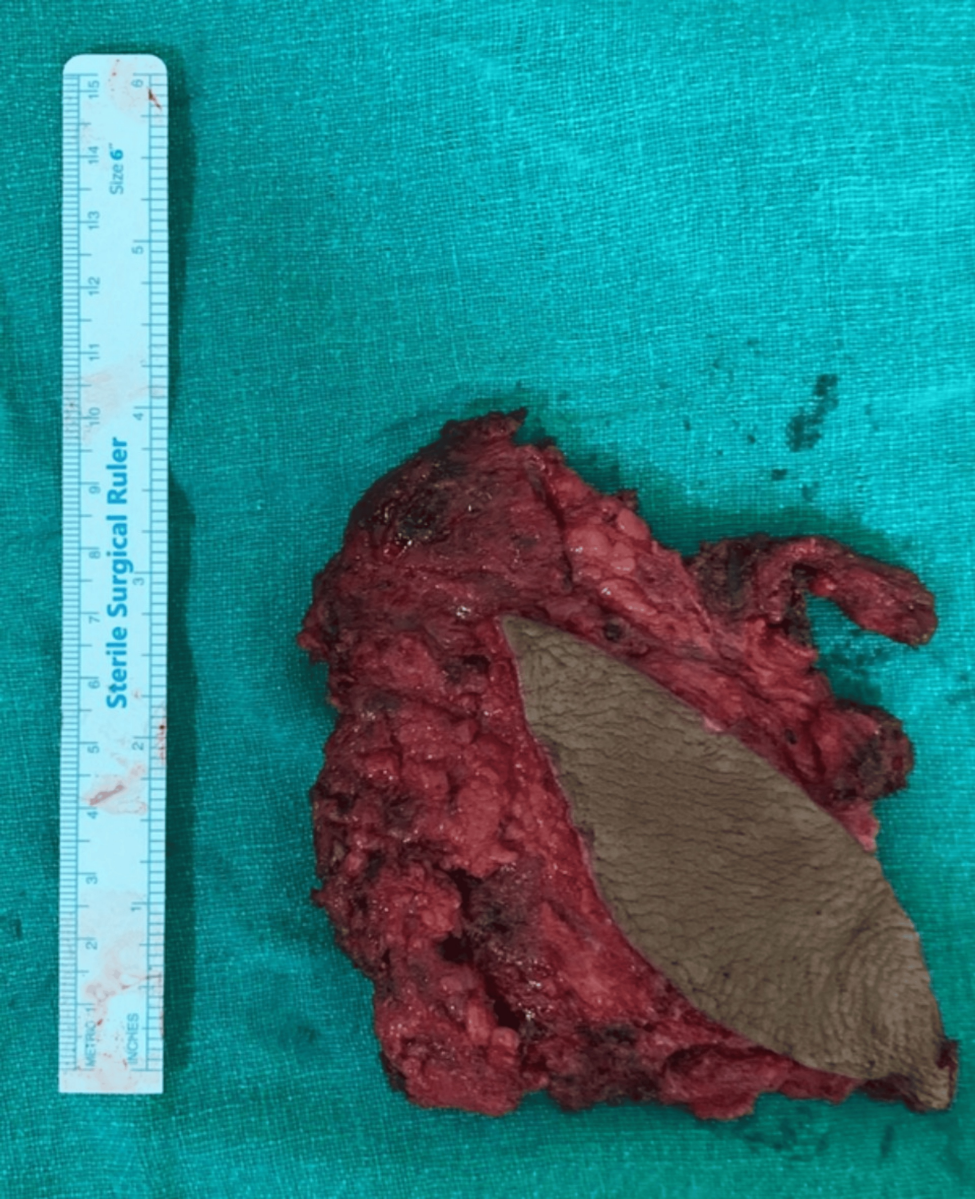
Resected AVM specimen AVM: arteriovenous malformation

One week later, the patient underwent surgical excision of the AVM under general anesthesia. Through a right posterolateral incision, the lesion was dissected and resected completely with preservation of the surrounding ribs and pleura. Estimated blood loss was approximately 300 mL. The resected mass measured 13 × 12 × 8 cm (Figure 5). Histopathology confirmed an AVM with direct arteriovenous connections, fibrous stroma, and no evidence of malignancy.

The patient had a smooth postoperative recovery. On the fifth postoperative day, the patient was discharged. At six-month follow-up, he remained asymptomatic with no clinical or imaging evidence of recurrence.

## Discussion

AVMs arise due to developmental anomalies during vascular morphogenesis. They are composed of a nidus of vessels where arteries drain directly into veins, bypassing the capillary system, leading to high-flow dynamics and pressure-related changes. Although congenital in origin, AVMs can remain dormant for years before becoming clinically apparent due to hormonal influences, infection, trauma, or growth [1,7]. Our patient’s lesion remained stable during childhood but showed rapid growth in late adolescence, consistent with findings in previously reported cases [2].

Chest wall AVMs are particularly rare and pose unique challenges due to their proximity to vital thoracic structures and the potential for extensive collateral vascularization [3]. The diagnosis of AVMs relies heavily on imaging. MRI offers excellent soft tissue resolution and helps distinguish high- from low-flow lesions. T1- and T2-weighted sequences, particularly with fat suppression techniques like STIR, can identify the extent of involvement and flow characteristics. In our case, MRI revealed a large, hypervascular lesion confined to soft tissues without bony or pleural invasion.

CT angiography provides critical vascular mapping, identifying arterial feeders and venous drainage. Our patient’s lesion received arterial supply from multiple intercostal arteries, which were selectively embolized using PVA particles and gelfoam. This technique is a well-established strategy for preoperatively devascularizing high-flow AVMs, thereby reducing operative blood loss and facilitating surgical excision [5,6].

While embolization alone may achieve a temporary reduction in flow, it is rarely curative. Several studies have demonstrated high recurrence rates when embolization is used as a stand-alone therapy, sometimes exceeding 90% [1,6]. Therefore, definitive surgical excision is often required, particularly for large or symptomatic AVMs. The surgical challenge lies in navigating dense fibrosis, distorted anatomy, and potential intraoperative bleeding. Preoperative embolization significantly reduces these risks [7].

Our patient’s successful outcome was the result of precise imaging, staged embolization, and complete resection. Histopathological analysis confirmed a classical AVM without malignant transformation, consistent with other reported cases [8]. The postoperative course was smooth, with the patient discharged within a week and showing no signs of recurrence at six months.

This case supports the existing body of literature advocating a staged, multidisciplinary approach to managing large, high-flow AVMs. Moreover, it adds to the limited reports of posterior chest wall AVMs, emphasizing the need for individualized planning based on lesion anatomy and vascularity. It also underscores the importance of long-term follow-up, as recurrence is a known issue with incomplete excision or revascularization through collateral formation [2,4].

## Conclusions

Congenital high-flow AVMs of the chest wall are rare and potentially debilitating lesions. Accurate diagnosis through MRI and CT angiography is essential to determine lesion extent and plan therapy. A staged approach combining embolization and surgical excision offers the best chance of complete removal and minimal complications. Our case demonstrates that with coordinated multidisciplinary care, excellent outcomes can be achieved in such challenging vascular anomalies.
